# The Story of the Insane from Year to Year

**Published:** 1894-08-25

**Authors:** 


### THE STORY OF THE INSANE FROM YEAR TO YEAR.

(Seventh Series.)

### CORNWALL COUNTY ASYLUM, BODMIN.

On January 1st, 1893, this asylum contained 697 patients, and by the end of the year the number had risen to 714. The admissions were 144, the recoveries 48, and the deaths 69. Table X., which gives the causes of insanity in those admitted, tells us that only one patient owed his disease to alcoholic excess, and this is the lowest proportion we have ever met with. Hereditary tendency is credited with 11, and previous attacks with 33, while in 21 cases no cause was ascertained. Influenza is put down as the cause in six and sunstroke in four. Of the deaths, 20 were from some form of brain disease and nine from consumption. Calculated on the admissions, the percentage of recoveries is 35·03, and the deaths, calculated on the average number resident work out at 9·8. The asylum is almost full, and 16 patients are boarded out in the Plymouth Asylum, where they must cost considerably more than they would do in their own asylum. Dr. Adams points out the necessity of providing accommodation for the insane poor of the county; and one of the most remarkable facts in connection with lunacy administration is the all but invariable tendency of committees to put off building as long as they can, forgetting that in the meantime much money has been spent in boarding out which might just as well have been spent in building. The weekly rate per head is 10s.

### EAST RIDING ASYLUM, BEVERLEY.

An increase of nine patients has to be noted, there being 317 in residence on the last day of the year. There were 74 admissions, 27 recoveries, and 27 deaths. These numbers show that the percentage of recoveries was 39·13, and that of the deaths 8·57. Hereditary tendency caused the insanity in 25 of the admissions, and intemperance in drink in 8. Brain diseases caused death in seven cases, and consumption in six. Dr. Macleod discusses the question of increase of insanity in the country, and he leans to the idea that it does not increase more than can be accounted for by the increase of the population. This is cheering, but he thinks that the nature of cases sent to our county asylums will tend to lower recovery rates, and so cause an accumulation of the insane. The committee say "the asylum maintains a high standard of excellence, and that the conduct of the officers and servants has been entirely creditable to them, and satisfactory to the committee."

### HANTS COUNTY ASYLUM, FAREHAM.

During the year 1893 the number of inmates increased from 1,006 to 1,020. There were 235 admissions, 88 recoveries, and 101 deaths. The percentage of recoveries stands at 37·4, and the deaths at 9·9. Hereditary tendency accounts for 68 of the year's admissions, and drink for 36. Previous attack is the assigned cause in 43 cases, and in 42 no cause was known. Thirty-nine of the deaths were due to brain diseases, and of these not fewer than thirteen were due to general paralysis. We are glad to note that Dr. Worthington gives this disease its proper name of "progressive paralysis," and it would be much better if all medical superintendents dropped the old name, which is never properly descriptive, and often incorrect. Sixteen of the deaths were caused by consumption. There has been some little overcrowding during the year; but it will be temporary only, and entirely removed with the opening of the new asylum for the Isle of Wight. The question of providing for the care and treatment of idiot children has been definitely settled by the erection of a block for idiot children. Dr. Worthington says it is on much the same lines as that attached to the Northampton County Asylum, which has proved so successful. There were two cases of typhoid, and these occurred in spite of much money previously spent on sanitary improvements and new waterworks. Dr. Worthington cannot assign any cause for the outbreak, and in this inability he is by no means singular, as a perusal of our county asylum reports conclusively shows. As we have more than once observed in these notices, a disease clinically identical with typhoid occasionally breaks out where there is no reason to suspect either sanitary imperfection or importation of the disease. We heartily congratulate Attendant John Phillips and Attendant John Williams on having obtained their well-earned pensions. The Committee of Visitors say that "much credit is due to the superintendent for his able management." The weekly rate of maintenance is a fraction under 9s. per head.

### COUNTY ASYLUM AT STAFFORD.

The number rose from 848 to 893. There were 343 admissions, 81 recoveries, and 108 deaths. The recoveries stand at 24·62, and the deaths at 12·54; the former being, as usual, calculated on the admissions, and the latter on the average number resident. Intemperance in drink is given as the cause of insanity in 66 of the year's admissions, and hereditary influences in 65, while 80 are put down as "unknown;" but if the causes in these could be ascertained there is no doubt that the hereditary and drink causes would have to be very materially increased. The lunacy laws fill a big octavo volume, but one clause forbidding insane persons to marry, and another to prevent people driving themselves mad with drink would be worth the whole volume. Brain diseases were the causes of death in 42 cases, and consumption in 24. The asylum accommodation is totally insufficient for the county, and a new asylum is to be built. In the meantime 228 patients are boarded out in other asylums, and it may be reasonably assumed that the new asylum will not be opened within four years, these 228 patients will have cost the ratepayers of Stafford something like £10,000 over and above what they would have cost if the wants of the county had been anticipated. A hospital for infectious diseases is to be built at an estimated expense of £3,500; and Dr. Christie says that several important improvements have been carried out during the year. The committee bear "testimony to the able management of the asylum by the medical superintendent." The weekly cost is 9s. 3¾d.

### TIPPERARY ASYLUM AT CLONMEL.

There is a custom among Irish asylums which we should like to see followed in England, namely, that of giving the accommodation; but with the number of patients should also be given the cubic space allotted to each. Here we find the accommodation stated to be intended for 600 patients, and there are 636 in residence, being an increase of 15 during the year. The admissions were 113, the recoveries 43, and the deaths 40. The recoveries yield a percentage of 38 on the admissions, and the deaths of six on the average number resident. Thirty of the admissions were owing to hereditary influence and seven to drink. The mortality from consumption was very high; in fact, this disease carried off ten of the total of forty deaths. Dr. Garner goes carefully and fully into the question of increase of insanity, and he records his opinion that "without taking an unduly optimistic view, it may be deduced that the apparent increase is due, amongst other causes, rather to the survival of old cases, through the prolongation of life, than to any increase of occurring cases."

Dr. Garner is quite justified in congratulating himself that no serious accident occurred during the year, and he adds that "the consciousness is ever present that we who have charge of such unstable elements are living, as it were, on the verge of a volcano, and subject to constant alarms. Accordingly life inside these walls is not altogether rose-coloured." This is too true. Verily a medical superintendent's life is not a happy one. The annual cost per head is £23 3s. 6d.

